# Application of a practical methodology for the selection of suitable value chains to produce circular fertilisers from secondary raw materials

**DOI:** 10.12688/openreseurope.19506.1

**Published:** 2025-02-06

**Authors:** Lidia Paredes, Elisa Gambuzzi, Rita Gentili, Jessica Pérez-García, Ambrogio Pigoli, Inès Verleden, Pedro Villanueva-Rey, Werner Vogt-Kaute, Wim Moerman, Lucía González-Monjardin

**Affiliations:** 1 Galician Water Research Center Foundation (Cetaqua Galicia), AquaHub - A Vila da Auga, Rúa José Villar 6 Granjel 33, Santiago de Compostela, 15890, Spain; 2 Technology Centre for Energy and the Environment (CETENMA), C. Sofía, 6, 13, Cartagena, 30353, Spain; 3 Coldiretti, Via XXIV Maggio n. 43. Roma, 00187, Italy; 4 Italian Composting and Biogas Consortium (CIC), Via Dalmazia 2, Treviglio, 240247, Italy; 5 Inagro, Ieperseweg 87, Rumbeke-Beitem, 8800, Belgium; 6 Naturland e.V, Kleinhaderner Weg 1, Gräfelfing, 82166, Germany; 7 GreenTile BV, Harelbeekstraat 104D, Zwevegem, 8550, Belgium

**Keywords:** Agriculture, bio-waste, circular fertilisers, compost, organic by-products, resource efficiency, sewage sludge, wastewater

## Abstract

**Background:**

The growing demand for food products, driven by a growing world population, has increased Europe's dependence on conventional fertilizers, which have a high impact on the environment. In the last decade, new circular fertilizer value chains have appeared as promising alternatives to conventional fertilizers.

**Methods:**

Because of the huge number of alternatives, this study aimed to develop a practical methodology that facilitates the analysis of data related to each value chain to identify and select the most promising circular fertilizer value chains to promote their wide-scale production and use in agriculture, replacing the dependence on conventional fertilizers in Europe. This methodology is based on two stages (funnelling process and scoring system) and considers the 16 criteria defined in the study. The methodology was tested for 48 value chains identified during the mapping of secondary raw materials in Europe with the potential to be used as circular fertilizers when processed, classifying them into seven different raw materials: urban wastewater (UWW), industrial wastewater (IWW), sewage sludge (SS), biowaste (BW), biological by-products (BBP), treated manure (TM), and digestate (DIG). The funnelling process is based on a GO/NO-GO approach that meets six criteria and allows the discarding of 18 value chains, from 30 to the second stage. The scoring system was a more complete analysis, including ten new scoring criteria.

**Results:**

This system allowed the identification of the potential of the value chains analyzed, concluding that struvite from UWW, struvite from IWW, stabilized sludge from SS, composted biowaste from BW, feather meal from BBP, solid fraction from DIG, and spent mushroom substrate from TM are the most promising options for agriculture.

**Conclusions:**

The develop methodology was used to evaluate 48 different value chains with the potential to generate promising circular fertlizers. Seven value chains were finally selected.

## 1. Introduction

The application of fertilizers in agricultural fields is essential to ensure food production and safety. World population growth is expected to increase food demand by more than 50% by 2050. Consequently, the demand for fertilizer products is expected to increase proportionally to guarantee the availability of food. Fertilizer products contain primary macronutrients such as nitrogen (N), phosphorus (P), and potassium (K); secondary macronutrients such as calcium (Ca), magnesium (Mg), and sulfur (S); or a combination of both, which are required to provide the nutrients that plants need and to increase soil fertility and crop productivity.

Conventional fertilizers are produced through synthetic and manufacturing processes from raw materials such as air, natural gas, and mined ores. The application of conventional fertilizers is very extended in the European Union (EU) since they are used on 75% of agricultural land, and their application is expected to increase globally at a rate of +2% in the next few years ( European Commission, 2019a). The EU is largely dependent on imports of most conventional fertilizers and their main macronutrients (N, P, K), which come from finite and non-renewable sources located in third countries. Additionally, the use of conventional fertilizers has associated harmful environmental impacts due to the methods used for the extraction of nutrients, manufacturing processes, and significant CO _2_ emissions associated with the transport of fertilizer products to other countries different from the production regions ( Basosi *et al.*, 2014).

Among the EU’s political priorities, the replacement of conventional fertilizers by circular fertilizers produced from locally available secondary raw materials, such as wastewater and biowaste, is prioritized and promoted. An important initiative to support this goal is the new EU Fertilising Products Regulation that went into force in 2022 (Regulation EU 2019/1009) ( European Commission, 2019b), which establishes the conditions to make use of recycled or organic materials for fertilizing purposes and introduces them in the EU market. According to data provided by the European Commission ( European Commission, 2016), it is estimated that recycled biowaste could substitute up to 30% of conventional fertilizers, whereas the recovery of phosphorus from sewage sludge, biodegradable waste, meat and bone meal, or manure could replace up to two million tons of the six million tons of phosphate rocks that are imported every year by the EU. The application of circular fertilizers in agriculture has several benefits since their use improves soil structure, carbon storage, water, and nutrient retention, as well as reduces the carbon footprint compared with the production of conventional fertilizers ( Culas *et al.*, 2025; Lam *et al.*, 2020). 

Multiple value chains for circular fertilizers have appeared in the last decade as promising alternatives to replace the use of conventional fertilizers. These value chains differ in the input secondary raw materials (e.g., wastewater, biowaste, manure), production processes based on nutrient recovery technologies (e.g., composting, anaerobic digestion, precipitation, stripping), and output circular fertilizers (e.g., compost, struvite, ammonium sulfate, phosphates, etc.). Nevertheless, all of them allow the matching of the availability of valuable nutrients contained in the different secondary raw materials with the necessity of using circular fertilizers to replace the conventional ones, to boost the circular economy concept, and to increase the sustainability of industrial processes. Some of these value chains are already well established and the circular fertilizers market-available (e.g., compost production from biowaste) ( Czekała *et al.*, 2017; Toledo *et al.*, 2020), whereas others are still under research because of low technology readiness level (TRL) (e.g., vivianite production from urban wastewater) ( Wilfert *et al.*, 2018; Wu *et al.*, 2019) or their commercialization is hindered due to legal barriers (e.g., struvite production from urban wastewater) ( de Boer *et al.*, 2018). Despite the huge number of options found in the literature related to circular fertilizer value chains, no specific methodologies have been identified in other studies that can contribute to identifying and selecting the most promising circular fertilizers to replace the use of conventional fertilizers in the EU.

In this sense, in the framework of the FER-PLAY project (a Horizon Europe funded project), a database was created to gather and harmonize the existing knowledge on key circular fertilizer value chains produced in the EU from seven secondary raw materials (urban wastewater (UWW), industrial wastewater (IWW), sewage sludge (SS), biowaste (BW), biological by-products (BBP), treated manure (TM), and digestate (DIG), and a practical methodology based on two stages (funnelling process and scoring system) was developed to identify and select the most promising ones for promoting their wide-scale production and use in agricultural fields, taking into consideration 16 criteria defined in this study, which include, among others, secondary raw material availability, technical viability, nutrient content, type of transport and ease, and type of storage and ease.

## 2. Methods

### 2.1. Mapping and selection of circular fertiliser value chains

A complete database (freely accessible online, https://doi.org/10.5281/zenodo.11060654) was created, including the most important characteristics of several circular fertilizer value chains, classifying all of them into seven different raw secondary materials: urban wastewater (UWW), industrial wastewater (IWW), sewage sludge (SS), biowaste (BW), biological by-products (BBP), digestate (DIG) and treated manure (TM). The list of output products ( Table 1) includes struvite, vivianite, K-struvite, phosphates, stabilized sludge, compost, P-rich ashes, ammonium sulfate, ammonium nitrate, biochar, hydrochar, mineral concentrate, spent mushroom substrate, liquid fraction of digestate, solid fraction of digestate, hair powder pellets, and horngrits/homchips. At the time of the selection process, 48 value chains were mapped, but the database is constantly updated and currently includes 51 value chains. The value chains were selected based on a mapping conducted by the partners of the FER-PLAY project, which integrates circular fertilizer producers, farmer associations, and R&D centers with experience in circular bioeconomy. The following sources were considered for the mapping: the selection of circular fertilizer value chains and data collection related to each value chain: i) know-how of partners’ and partners’ extensive networks; ii) peer-reviewed scientific publications; iii) past and current projects and initiatives developed under the EU funding programs relevant to the topic; iv) databases; v) statistics and market studies; and vi) external stakeholders linked to each value chain.

**Table 1.** Preselection of the main circular fertiliser value chains from secondary raw materials.

Raw material	Fertiliser	References
Urban wastewater (UWW)	Struvite	Arcas-Pilz *et al.* (2021)
	Vivianite	Wilfert *et al.* (2018)
	K-struvite	Data not published
	Phosphates	Eureau (2021)
	Stabilized sludge	Zilio *et al.* (2022)
Industrial wastewater (IWW)	Struvite	Ylmén *et al.* (2018)
	Vivianite	Wu *et al.* (2019)
	K-struvite	Data not published
Sewage sludge (SS)	Struvite	Ferraro *et al.* (2023)
	Vivianite	Wijdeveld *et al.* (2022)
	Phosphates	Data not published
	Composted sewage sludge	Bożym & Siemiątkowski (2018)
Biowaste (BW)	Composted biowaste (green compost)	Anceschi *et al.* (2018)
	Composted biowaste (food waste and green compost)	Torrijos *et al.* (2021)
	Struvite	Siciliano (2016)
	P-rich ashes	Luyckx & Caneghem (2022)
	Biochar	Nisticò *et al.* (2019)
	Hydrochar	Taskin *et al.* (2019)
Biological by- products (BBP)	Composted biological by-products	Toledo *et al.* (2020)
	Hair powered pellets	Data not published
	Feather meal	Gerber *et al.* (1999)
	Horngrit/homchips	Data not published
	Meat-, Meatbonemeal	Bøen & Haraldsen (2013)
Digestate (DIG)	Untreated (raw) digestate with animal manure	Samoraj *et al.* (2022)
	Untreated (raw) digestate without animal manure (plant- based)	Van Puffelen *et al.* (2022)
	Liquid fraction of digestate	Tambone *et al.* (2017)
	Solid fraction of digestate	Tambone *et al.* (2017)
	Composted digestate	Czekała *et al.* (2017)
	Struvite	Szymanska *et al.* (2019)
	Vivianite	Wilfert *et al.* (2018)
	K-struvite	European Commission: Joint Research Centre *et al.* (2019)
	Phosphates	European Commission: Joint Research Centre *et al.* (2019)
	P-rich ashes	Hämäläinen *et al.* (2021)
	Enriched biosolids with struvite	Data not published
	*Tenebrio molitor frass*	Houben *et al.* (2021)
Treated manure (TM)	Composted animal manure	Vukobratović *et al.* (2018)
	Ammonium nitrate	Samanta *et al.* (2022)
	Ammonium sulphate	Menkveld & Broeders (2017)
	Mineral concentrate	Cempa *et al.* (2022)
	Vivianite	European Commission: Joint Research Centre *et al.* (2019)
	Phosphates	European Commission: Joint Research Centre *et al.* (2019)
	Biochar	Data not published
	Hydrochar	Fu *et al.* (2022)
	Solid fraction of manure	Fernández-Rodríguez *et al.* (2021)
	Liquid fraction of manure	Sigurnjak *et al.* (2017)
	K-struvite	Company *et al.* (2022)
	Spent mushroom substrate	Tuhy *et al.* (2015)
	Manure-processing effluent	Data not published

For each value chain selected, the FER-PLAY database (freely accessible online, https://doi.org/10.5281/zenodo.11060654) includes information related to: i) production (including process description, raw material availability, volume produced, number of production plants, Technology Readiness Level (TRL)), ii) distribution/trade (including national/international demand, transport area, transport ease, import, export), iii) storage and application (storage ease, storage necessities, storage emissions, pH, uptake speed, application form, application type, application timing, application dose, application emissions), iv) product content (including nitrogen, phosphorus, potassium, organic matter, sulphur, moisture content, calcium, magnesium, other micronutrients, heavy metals, pharmaceuticals, pathogens, microplastics), v) cost (including CAPEX, OPEX and market price) and vi) legislation (including applicability in organic farming, possibility of application in nitrate vulnerable zones (NVZ), national regulatory framework, product function category (PFC) and component material category (CMC) according to the Regulation EU 1009/2019)).

### 2.2. Funnelling process

Several circular fertilizers are available in the market or are close to being marketed. To ensure geographic representativity, coverage, and replicability multi-assessment, it was crucial to assess only those value chains that are well characterized and represent the variability of agricultural applications and practices. To this end, a funnelling process based on a GO/NO-GO approach was applied. The funnelling process facilitates:

- The selection of relevant circular fertilizer value chains from a multitude of existing value chains so that further impact assessment is feasible and resource effective.
- Quickly disregard those value chains that are not properly characterized or not viable for industrialization and/or their application due to various problems (e.g., low nutrient content, toxicity, technical nonviability), not considering them for the assessment phase.

**Figure 1.**
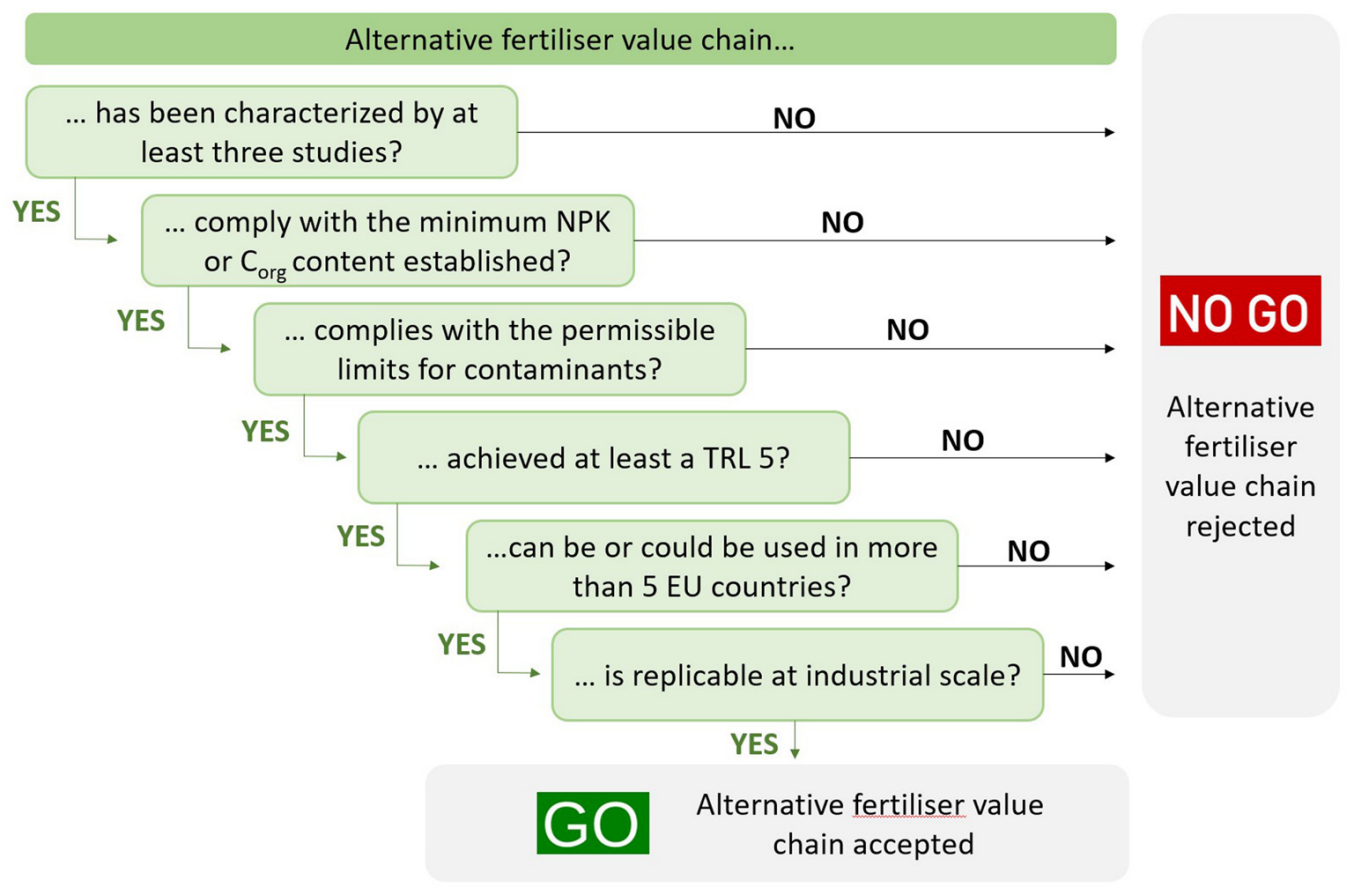
GO/NO GO approach for the evaluation of each value chain.

The GO/NO GO approach ( Figure 1) is based on the application of a set of six predefined criteria. Every value chain was examined against the first criterion, and if the result was positive (GO), it was analyzed against the following and so on: Only those value chains that passed all stages obtained the “GO” label and were considered as promising and subjected to further assessment through the scoring system. After applying the funnelling process to the 48 circular fertilizer value chains, the obtained results were reviewed to ensure consistency. The value chains that seemed promising and received the “NO GO” label were scrutinized in detail. To accept the inclusion of these circular fertilizers, they must be widely used in Europe because their use demonstrates their capacity as a circular fertilizer in addition to their advanced development of production technology.

Six criteria were considered during the funnelling process to classify the GO/NO GO. These criteria cover different aspects of circular fertilizer value chains. Their definitions are as follows:

- **C1**. Data availability. At least three different studies have characterized the value chain.
- **C2**. Nutrient and organic carbon content. C2 was split into two categories: 2(A)-Nutrient content and 2(B)-Soil improvement capacity. On the one hand, the circular fertilizer (linked to category 2(A)) presented the minimum NPK content established by one of the possible PFC according to Regulation EU 2019/1009. On the other hand, the circular soil improver (linked to category 2(B)) presents the minimum organic carbon (C _org_) content established by one of the possible PFC from the classification according to Regulation EU 2019/1009.
- **C3**. Toxicity. The circular fertilizer does not exceed the maximum permissible limits for contaminants (e.g., heavy metals, such as cadmium, arsenic, chromium, and mercury; microorganisms, such as *Salmonella spp* and *Escherichia coli*; and pharmaceuticals) established in Regulation EU 2019/1009.
- **C4**. Technical viability. The technology used for the recovery of nutrients in the value chain achieved a Technology Readiness Level (TRL) of at least five.
- **C5**. Versatility. The circular fertilizer can or could be used in more than five countries according to their national legislation and/or comply with Regulation EU 2019/1009. Due to the difficulty of accessing and interpreting the national/regional laws of the different countries, this criterion mainly uses Regulation EU 2019/1009 as a reference.
- **C6**. Industrialization. The circular fertilizer value chain is replicable at the industrial level around the EU, that is, feedstocks that are common in the EU.

For the evaluation of the criteria based on the European Fertilizing Regulation (C2, C3, and C5), at least one PFC was designed for each of the analyzed circular fertilizer value chains. For this classification, the NPK and C _org_ contents assigned in the FER-PLAY database for each fertilizer were considered since they are the main parameters linked to the nutrient content and soil amendment capacity. Compliance with the requirements of any of the CMC for the raw materials from which the fertilizer was produced was excluded from the study. The absence of a PFC assignment directly indicates that C2 has not been fulfilled. Table 2 summarizes the PFC classifications related to the 48 value chains evaluated during the funnelling process.

**Table 2.** Product Function Category (PFC) classification for circular fertiliser value chains evaluated during the funnelling process.

Raw material	Fertiliser	PFC
Urban wastewater (UWW)	Struvite	1.C.I.a.i
	Vivianite	1.C.I.a.i
	K-struvite	-
	Phosphates	-
	Stabilized sludge	3.A
Industrial wastewater (IWW)	Struvite	1.C.I.a.i
	Vivianite	1.C.I.a.i
	K-struvite	1.C.I.a.i
Sewage sludge (SS)	Struvite	1.C.I.a.i
	Vivianite	1.C.I.a.i
	Phosphates	1.C.I.a.i
	Composted sewage sludge	3.A
Biowaste (BW)	Composted biowaste (green compost)	3.A
	Composted biowaste (food waste and green compost)	3.A
	Struvite	1.C.I.a.i
	P-rich ashes	1.C.I.a.i
	Biochar	1.A.I./3.A/3.B
	Hydrochar	1.A.I./3.A/3.B
Biological by- products (BBP)	Composted biological by- products	3.A
	Hair powered pellets	1.A.I/1.C.I.a.i/3.A
	Feather meal	1.A.I/1.C.I.a.i/3.A
	Horngrit/homchips	1.A.I/1.C.I.a.i/3.A
	Meat-, Meatbonemeal	1.A.I
Digestate (DIG)	Untreated (raw) digestate with animal manure	-
	Untreated (raw) digestate without animal manure (plant- based)	-
	Liquid fraction of digestate	-
	Solid fraction of digestate	3.A
	Composted digestate	3.A
	Struvite	1.C.I.a.i
	Vivianite	1.C.I.a.i
	K-struvite	-
	Phosphates	-
	P-rich ashes	1.C.I.a.i
	Enriched biosolids with struvite	-
	*Tenebrio molitor frass*	1.A.I
Treated manure (TM)	Composted animal manure	3.A
	Ammonium nitrate	1.C.I.b.i
	Ammonium sulphate	1.C.I.b.i
	Mineral concentrate	1.C.I.b.i
	Vivianite	1.C.I.a.i
	Phosphates	-
	Biochar	3.A
	Hydrochar	3.A
	Solid fraction of manure	3.A
	Liquid fraction of manure	-
	K-struvite	1.C.I.a.i
	Spent mushroom substrate	3.A
	Manure-processing effluent	-

*The symbol (-) indicates that the parameters to establish PFC are not fulfilled*.

### 2.3. Scoring system

Once the GO/NO-GO approach was applied, the circular fertilizer value chains that obtained the “GO” label were validated using a scoring system. The scoring system was based on the assignment of points under a set of ten defined categories. The scores ranged from 1 to 5, with 1 indicating “poor compliance” with the criteria, and 5 showing “excellent” compliance. The criteria defined for the application of the scoring system are described below. Table 3 summarizes the requirements established for the assignment of values from 1 to 5 corresponding to each scoring criterion.

**Table 3.** Criteria for the scoring system of circular fertiliser value chains.

Criteria	1	2	3	4	5
**SC1**	The lower abundancy	75% less ref	50% less ref	25% less ref	The highest abundane (ref)
**SC2**	The lower references	75% less ref	50% less ref	25% less ref	The highest references (ref)
**SC3**	TRL 5	TRL 6	TRL 7	TRL 8	TRL 9
**SC4**	The lower production	75% less ref	50% less ref	25% less ref	The highest production (ref)
**SC5**	-	Not marketable	-	Marketable	-
**SC6**	Liquid or frozen or solid, flammable and toxic	Liquid or frozen or solid, flammable or toxic	Liquid or frozen, no chemical hazards	Solid, refrigerated	Solid, no temperature requirements
**SC7**	Liquid or frozen or solid, flammable and toxic	Liquid or frozen or solid, flammable or toxic or emissions	Liquid or frozen, no chemical hazards	Solid, refrigerated	Solid, no temperature requirements
**SC8**	Manually	Mechanically located	Mechanically (located and random)	Compatible with existing technologies or possible of automation- mechanically	Compatible with existing technologies and possibility of automation
**SC9**	-	Use not allowed	-	Use allowed	-
**SC10**	-	Manure content	-	No manure content	-

*The abbreviation (ref) is referred to the list of references used for the creation of the database.*

- **SC1**. The secondary raw material is abundant across Europe. The abundances of the seven raw materials in Europe were classified based on the available information included in the FER-PLAY database. The raw material with the highest amount of annual production in Europe received a score of 5, whereas the other raw materials received a score based on the percentage of abundance compared to the most abundant raw material ( Table 3). In the case of the production of biological by-products, only production data from Germany was available because of the inaccessibility of the data.
- **SC2**. References on nutrient recovery technology across Europe. To determine the level of study and knowledge of the technology and fertilizer evaluated, a bibliographic search was conducted using Scopus ( https://www.scopus.com). A bibliographic search was carried out for each fertilizer evaluated according to their keywords. The search was limited to studies conducted in Europe over the last ten years in Europe. A total of 641 scientific articles were found on the 30 circular fertilizers evaluated at this stage. The fertilizer with the highest number of references received a score of 5, whereas the others received a score based on the percentage of the number of references compared to the most referenced ( Table 3).
- **SC3**. Production maturity across Europe. The scoring for this criterion was based on the TRL established by the EU (TRL EU). The TRL varied from 5 (1 point) (the minimum requirement to reach this scoring stage) to 9, the maximum existing TRL, and therefore, the maximum score (5 points). The TRL data for each fertilizer were defined based on the information gathered during the creation of the FER-PLAY database.
- **SC4**. The forecasted volume of circular fertilizer is significant across Europe. This criterion is based on the expected volume of production for each fertilizer included in the FER-PLAY database. Considering that there is not an expected production value for Europe for all circular fertilizers, the available data corresponding to each country were extrapolated to the European area based on the national production and area of each country. The fertilizer with the highest forecasted volume of annual production received a score of 5, whereas the other fertilizers received a score based on the percentage of the forecasted volume compared to the most abundant ( Table 3).
- **SC5**. Marketability of circular fertilizers across Europe. This criterion is based on the EU Fertilizing Products Regulation (Regulation EU 2019/1009). This regulation establishes rules for placing EU fertilizing products on the market. Therefore, those products that fulfilled the requirements of this legislation received a score of 4 points, whereas those that did not fulfil them received a score of only 2 points.
- **SC6**. Circular fertilizer transport and ease. The characteristics of the fertilizer for its transport in terms of the state of the fertilizer (liquid, frozen, or solid), chemical risks (flammable or toxic), and temperature requirements were evaluated, as shown in Table 3.
- **SC7**. Circular fertilizer type of storage and ease. The characteristics of the fertilizer for its storage in terms of the state of the matter (solid, liquid, or frozen), chemical risks (flammable, toxic, or emissions), and temperature requirements are shown in Table 3.
- **SC8**. Circular fertilizer application in agricultural fields. The ease of fertilizer application was evaluated using this criterion. Fertilizers that must be applied manually received the lowest score (1 point), whereas those with existing machinery with the possibility of mechanical automation received the highest score (5 points). Intermediate scores are presented in Table 3.
- **SC9**. Circular fertilizer is used for organic production. This criterion is based on EU Regulation EU 2021/1165, which authorizes certain products and substances for their use in organic production. Therefore, all fertilizers in the list received a score of 4 points, whereas those not in the list received only two points.
- **SC10**. Nitrogen content in circular fertilizers. The NVZ are geographical areas designated by governments to protect water resources from nitrogen pollution. Excess nitrogen from agricultural sources is one of the main causes of water pollution in Europe. The designation of NVZ is intended to prevent the overapplication of nitrogen-based fertilizers, which can result in excessive nutrient runoff and leaching. In EU countries, farmers within designated NVZ are required to fulfil national regulations regarding the application of nitrogen as fertilizer from manure (such as limits on the amount that can be applied and the timing). Due to the great difficulty in accessing these national regulations as well as the variety of conditions depending on the categorization of the areas, it was considered to "penalize" fertilizers obtained from manure. Consequently, all fertilizers that contained or came from animal manure were scored with two points since their application in the NVZ will always be limited (either by quantity or by season). The other fertilizers obtained a score of three points.

## 3. Results and discussion

### 3.1. Classification based on the funnelling process

**Figure 2.**
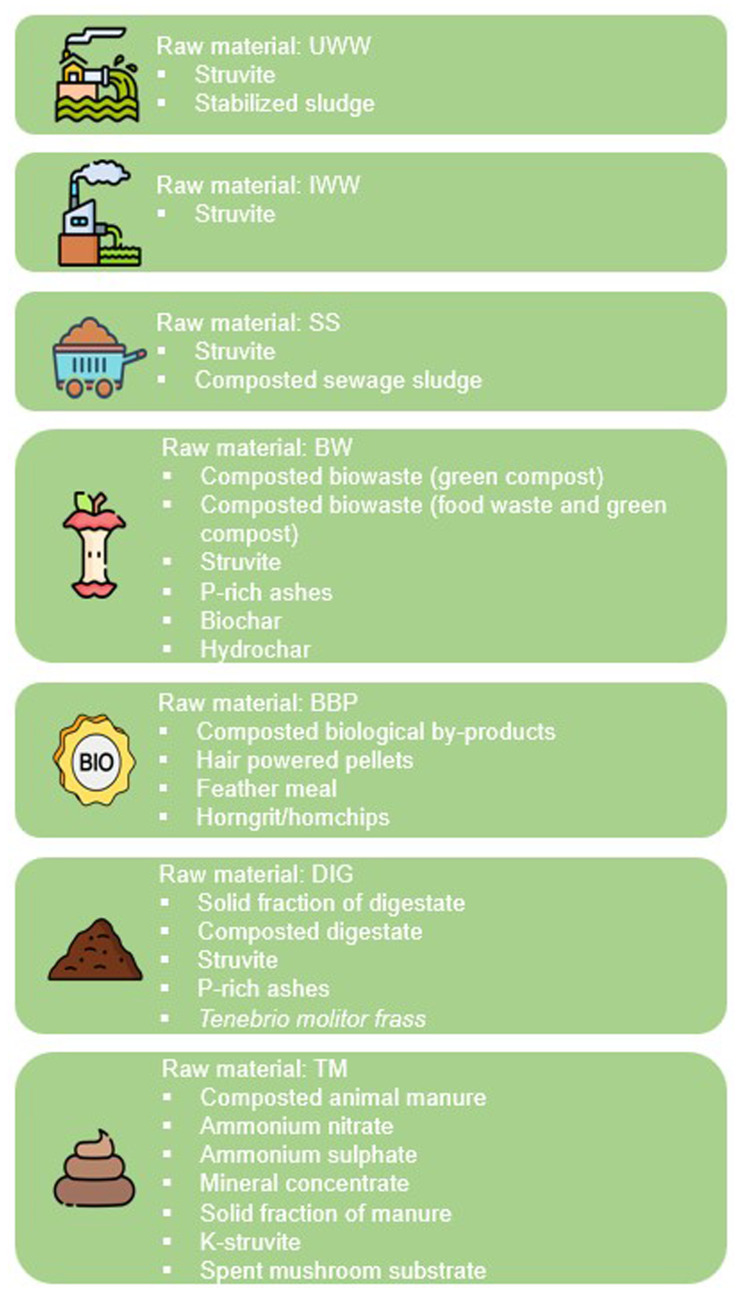
Value chains classified with the “GO” label after the funnelling process.

Out of a total of 48 circular fertilizer value chains evaluated during the funnelling process ( Table 1), 27 obtained a favorable classification of "GO" ( Figure 2). Consequently, they passed directly to the second stage of assessment through the scoring system. In contrast, 23 were classified as "NO GO" and were initially discarded. Table 4 summarizes the circular fertilizer value chains initially discarded, as well as the criteria and reasons for which they were discarded.

**Table 4.** Value chains classified with the “NO GO” label after the funnelling process.

Raw material	Fertiliser	Critical criteria	Reasons
Urban wastewater (UWW)	Vivianite	C3-Toxicity	No qualitative data found
		C4-Technical viability	TRL 3 (TRL < 5)
		C6-Industrialization	More development is needed
	K-struvite	C2(A)-Nutrient content	P2O5 < 12%
		C3-Toxicity	No qualitative data found
		C4-Technical viability	TRL 4 (TRL < 5)
		C5-Versatility	Does not comply with EU regulation and could not be classified under any PFC
		C6-Industrialization	More development is needed
	Phosphates	C2(A)-Nutrient content	No qualitative data found
		C3-Toxicity	No qualitative data found
		C4-Technical viability	TRL 3–4 (TRL < 5)
		C5-Versatility	No qualitative data found
		C6-Industrialization	More development is needed
Industrial wastewater (IWW)	Vivianite	C3-Toxicity	No qualitative data found
		C4-Technical viability	TRL 4 (TRL < 5)
		C6-Industrialization	More development is needed
	K-struvite	C6-Industrialization	More development is needed
Sewage sludge (SS)	Vivianite	C3-Toxicity	No qualitative data found
		C6-Industrialization	More development is needed
	Phosphates	C4-Technical viability	TRL 3–4 (TRL < 5)
		C6-Industrialization	More development is needed
Biological by- products (BBP)	Meat-, Meat bone meal	C1-Data availability	Not enough data in studies
Digestate (DIG)	Untreated (raw) digestate with animal manure	C2(A)-Nutrient content or C2(B)- Soil improvement capacity	Cannot be classified under any PFC. Does not comply with any NPK & C _org_ requirements
	Untreated (raw) digestate without animal manure (plant-based)	C2(A)-Nutrient content or C2(B)- Soil improvement capacity	Cannot be classified under any PFC. Does not comply with any NPK & C _org_ requirements
	Liquid fraction of digestate	C2(A)-Nutrient content or C2(B)- Soil improvement capacity	Cannot be classified under any PFC. Does not comply with any NPK & C _org_ requirements
	Vivianite	C3-Toxicity	High potential of iron toxicity
		C6-Industrialization	More development is needed
	K-struvite	C2(A)-Nutrient content	No qualitative data found
	Phosphates	C2(A)-Nutrient content	No qualitative data found
	Enriched biosolids with struvite	C2(A)-Nutrient content	Cannot be classified under any PFC
		C5-Versatility	Not complied and do not have PFC classification
Treated manure (TM)	Vivianite	C3-Toxicity	High potential of iron toxicity
		C6-Industrialization	More development is needed
	Phosphates	C2(A)-Nutrient content	No qualitative data found
		C3-Toxicity	No qualitative data found
	Biochar	C3-Toxicity	No qualitative data found
	Hydrochar	C3-Toxicity	High potential of zinc and cadmium toxicity
	Liquid fraction of manure	C2(B)-Soil improvement capacity	Corg < 5%. Cannot be classified under any PFC. Does not comply with any NPK & Corg requirements
		C3-Toxicity	No data found in the database
		C5-Versatility	It could not comply with any PFC classification
	Manure-processing effluent	C2(B)-Soil improvement capacity	Corg < 5%. Cannot be classified under any PFC. Does not comply with any NPK & Corg requirements

Among the value chains that obtained the "GO" label, only struvite passed the funnelling process for fertilizers produced from IWW. Consequently, struvite obtained from industrial wastewater did not compete with other fertilizers within its own category during the application of the scoring system. For other secondary raw materials, such as UWW and SS, only two circular fertilizers passed favorably the funnelling process, whereas for the rest of the raw materials (BW, BBP, DIG and TM), the “GO” label was obtained for four to seven circular fertilizer value chains.

For the UWW raw material, the value chains that received the “NO GO” label were vivianite, K-struvite, and phosphates. They were discarded based on criterion C4 because the TRL did not exceed 4 for any of them. This low TRL directly leads to a lack of data that prevents the determination of their toxicity (C3) and, in some cases, their nutrient content (C2). Unfortunately, their low level of technological maturity means that their versatility (C5) and industrialization (C6) are not possible. In the specific case of vivianite, different bibliographic references have concluded that phosphorus recovery as vivianite is an innovative practice that is still in its infancy ( Wu *et al.*, 2019). The same pattern related to the level of immaturity of the nutrient recovery technology was detected for vivianite, K-struvite, and phosphates produced from IWW and SS.

Regarding the discarded value chain for BBP, meat bonemeal is a specific fertilizer produced in Germany. Although it was included within the 48 value chains initially selected because it is a promising option according to the literature ( Bøen & Haraldsen,, 2013; Sica *et al.*, 2025), not enough data are available at the European level to properly assess it as a promising circular fertilizer.

DIG was the raw material with the most "NO GO" labels, discarding seven value chains from the initial 12. The main reason for discarding was criterion C2. Untreated (raw) digestate with animal manure, untreated (raw) digestate without animal manure (plant-based), liquid fraction of digestate, K-struvite, phosphates, and enriched biosolids with struvite did not comply with any NPK and C _org_ requirements established for these fertilizers in Regulation EU 2019/1009. Therefore, they could not be classified within any PFC. Consequently, versatility (C5) was affected as well. In the case of vivianite, this value chain is discarded due to its high potential for iron toxicity (C3) ( European Commission: Joint Research Centre *et al.*, 2019).

In the case of TM, six circular fertilizer value chains were classified with the “NO GO” label. For vivianite and phosphates, the reasons are consistent with those described for DIG. In the case of biochar and hydrochar, the “NO GO” label was due to the toxicity criterion (C3). For biochar, no quantitative data were found, whereas for hydrochar, the values for zinc and cadmium content exceeded those established in the legislation. The liquid fraction of manure and manure-processing effluents could not be classified under any PFC because they did not comply with the NPK and C _org_ requirements established for C2.

Even though untreated (raw) digestate with animal manure, untreated (raw) digestate without animal manure, and the liquid fraction of digestate were classified as “NO GO” during the funnelling process, there are important reasons to consider their evaluation in the second assessment stage based on the scoring system. By one hand, according to data provided by the European Biogas Association ( EBA, 2024), the direct application of digestate as a circular fertilizer in agriculture is the most frequent destination for digestate in Europe (73%), followed by the use of upgraded digestate as a biofertilizer (15%). Some European countries, such as Sweden, have already designed voluntary schemes and guidelines for the application of digestates in soil. On the other hand, anaerobic digestion is booming in Europe owing to the demand for renewable gas, and digestate will be produced in increasing quantities in the upcoming years ( Bumharter *et al.*, 2023; EBA, 2024a). Therefore, this is considered a favorable context to take advantage of the potential use of digestate as a fertilizer. Additionally, several studies have demonstrated the advantages of using digestate as a circular fertilizer in agricultural fields. ( Karimi *et al.*, 2022) Long-term fertilization with digestate helps maintain soil fertility and support soil life, ensuring that high-yield agricultural lands can be sustainably utilized ( Karimi *et al.*, 2022). Several other benefits of digestate as an organic fertilizer are also worth highlighting. Compared to the same organic material in its raw form, digestate contains a higher percentage of readily available minerals, such as nitrogen, which enhances its fertilizing value. In addition to providing essential nutrients to plants, digestate offers further advantages over conventional agricultural fertilizers ( Czekała *et al.*, 2020).

### 3.2. Classification based on the scoring system

In total, 30 circular fertilizer value chains passed the funnelling process ( Section 3.1) and were evaluated through the application of the scoring system. Considering the criteria SC1-SC10 for each value chain and the corresponding score detailed in Table 3, Table 5 summarizes the score obtained for each fertilizer evaluated. The maximum score was obtained for the struvite produced from SS at 37 points.

**Table 5.** Scores obtained for each value chain after the assessment with the scoring system. In bold are the circular fertilisers with the higher score.

Raw material	Fertiliser	Score
UWW	**Struvite**	**36**
	Stabilized sludge	33
IWW	**Struvite**	**36**
SS	**Struvite**	**37**
	Composted sewage sludge	31
BW	Composted biowaste (green compost)	31
	**Composted biowaste (food waste and green compost)**	**33**
	Struvite	32
	P-rich ashes	24
	Biochar	26
	Hydrochar	27
BBP	**Composted biological by-products**	**28**
	Hair powered pellets	27
	Feather meal	27
	Horngrit/homchips	27
DIG	Untreated (raw) digestate with animal manure	28
	Untreated (raw) digestate without animal manure	27
	Liquid fraction of digestate	28
	Solid fraction of digestate	31
	Composted digestate	31
	**Struvite**	**34**
	P-rich ashes	26
	*Tenebrio molitor frass*	25
TM	Composted animal manure	33
	Ammonium nitrate	23
	Ammonium sulphate	23
	Mineral concentrate	24
	Solid fraction of manure	30
	K-struvite	21
	**Spent mushroom substrate**	**34**

For raw materials related to wastewater (UWW, IWW, and SS), the fertiliser with the highest score was struvite (36–37 points). Considering the triplicate representation of struvite in these categories, stabilized sludge was selected as one of the seven most promising fertilizers instead of struvite, as there were only three points of difference in their score for UWW (33 vs. 36). Additionally, considering that SS comes from urban wastewater, it was decided to consider the production of stabilized sludge from the SS raw material.

For fertilizers produced from BW, the composted biowaste (food waste and green compost) obtained the highest score with 33 points compared with the other five value chains evaluated.

The fertilizers produced from BBP raw materials obtained the lowest score compared to the rest of the value chains because of the lack of data for criterion SC4. Even though composted biological by-products were the fertilizer that achieved the highest score (28), considering that the composted BW was already selected as one of the seven most promising value chains to be assessed in the future based on a multi-assessment analysis, considering that there is only one point of difference with the other value chains evaluated for BBP, feather meal was selected as a promising option to be included in the list of the seven most promising value chains, taking into account its advantages compared with the other three value chains with the same score: i) this organic fertilizer is produced through controlled physical hydrolysis of fresh feathers, ii) its nitrogen content is equivalent to that of blood meal, but the nutrient release is slower, which is better for organic crops, and iii) higher availability of data for this value chain.

Regarding DIG raw material, struvite obtained the highest score of 34 points. Considering that struvite has already been represented previously as a promising fertilizer from UWW and IWW, solid fraction of digestate with a score of 31 points was selected as a promising fertilizer from DIG to be assessed in the future under a multi-assessment analysis. In the case of fertilizers produced from TM raw material, spent mushroom substrate obtained the highest score of 34 points.

The selection of the seven most promising fertilizers based on the FER-PLAY methodology are: struvite from UWW, struvite from IWW, stabilized sludge from SS, composted biowaste from BW, feather meal from BBP, solid fraction from DIG, and spent mushroom substrate from TM. This selection was conducted to ensure i) geographical representativeness, ii) availability of all raw materials and technologies in Europe, and iii) existence of the necessary machine for the application of each fertilizer in agriculture.

## 4. Conclusions

The practical methodology, developed in the framework of the FER-PLAY project and based on two stages (funnelling process and scoring system), allowed the identification and selection of the most promising circular fertilizer value chains out of a total of 48 included in the database created during the mapping of circular fertilizers in Europe, demonstrating that it is a useful tool that facilitates the analysis of data associated with the huge number of circular fertilizers that have appeared in the last decade as promising alternatives to conventional fertilizers.

The funnelling process, which is the first stage of the selection process, was used as a GO/NO GO approach to evaluate the 48 value chains based on six criteria that covered different aspects of the fertilizers, such as the composition, maturity of the production technology, and industrial replicability in different EU countries.

The scoring system, the second stage of the selection process, allowed the assessment of 30 circular fertilizer value chains based on the assignment of points under a set of ten defined categories covering different characteristics of the fertilizers, such as the availability of secondary raw materials across Europe, the marketability of the circular fertilizers across Europe, as well as practical aspects such as transport and storage of the fertilizer products and ease of use.

Combining the methodology developed in the framework of the FER-PLAY project with the specific interests of the stakeholders involved in the fertilizer value chain allowed the selection of one value chain for each raw material evaluated, with the seven most promising circular fertilizer value chains for Europe: struvite from UWW, struvite from IWW, stabilized sludge from SS, composted biowaste from BW, feather meal from BBP, solid fraction of digestate, and spent mushroom substrate from TM. The evaluation of the selected value chains in this study will be complemented through a multi-assessment analysis of their environmental, social, and economic impacts to promote their wide-scale production and use in agricultural fields in Europe.

## Data Availability

The FER-PLAY database is freely accessible online on Zenodo ( https://doi.org/10.5281/zenodo.11060654). ( Inagro (Belgium) *et al.*, 2024) Data are available under the terms of the Creative Commons Attribution 4.0 International license (CC-BY 4.0). Additional data for this article consists of bibliographic references, which are included in the References section.
