# Supplementary material for: Application of a practical methodology for the selection of suitable value chains to produce circular fertilisers from secondary raw materials
Source: Open Res Eur. 2025 Mar 31;5:43. Originally published 2025 Feb 6. [Version 2] doi: 10.12688/openreseurope.19506.2 (PMC11971626; doi:10.12688/openreseurope.19506.2)
Supplement: Peer review history

# Reviewer report: Reviewer response for version 2

**DOI:** 10.21956/openreseurope.21750.r52727

**Published:** 2025-05-27

**Authors:** Eduarda Fraga Olivo

**Affiliations:** 1 Universidade do Extremo Sul Catarinense (UNESC), Criciúma, Brazil

**Review Summary**

 The article presents a solid and innovative methodological proposal for the selection of suitable value chains for the production of circular fertilizers from secondary raw materials. The study addresses a highly relevant topic within the current context of the transition to a circular economy and more sustainable food systems. The methodology is well-structured and illustrated through empirical applications, which strengthens its practical replicability.

**Strengths**

 The proposed methodology is clearly described, with well-defined steps and comprehensive evaluation criteria (economic, environmental, social, and regulatory);

 The focus on waste valorisation and the production of circular fertilizers contributes directly to sustainability goals and the circular economy agenda;

 The practical application of the methodology to real-world case studies adds consistency to the proposal and demonstrates the feasibility of the approach in concrete scenarios.

**Weaknesses and Suggestions for Improvement**

 It is recommended to include a sensitivity analysis to assess the impact of varying the weights assigned to the evaluation criteria, which would enhance the robustness of the results;

 Although stakeholder involvement is mentioned, the extent to which their inputs influenced the evaluation and final selection of value chains could be described in more detail, including quantitative aspects;

 The economic analysis, although present, could be more thoroughly developed. The inclusion of financial indicators such as cost-benefit assessments of the prioritized solutions would strengthen the evaluation;

 I also suggest reviewing the English language in specific instances, particularly the use of “fertiliser” versus “fertilizer,” depending on whether the journal follows British or American English conventions.

Is the rationale for developing the new method (or application) clearly explained?

Partly

Is the description of the method technically sound?

Yes

Are the conclusions about the method and its performance adequately supported by the findings presented in the article?

Partly

If any results are presented, are all the source data underlying the results available to ensure full reproducibility?

Yes

Are sufficient details provided to allow replication of the method development and its use by others?

Yes

Reviewer Expertise:

As a graduate in Biological Sciences and a current Master's student in Materials Science and Engineering at UNESC, with a dual degree program in Mechanical Engineering, Energy and Materials at the Polytechnic Institute of Viana do Castelo (Portugal), my academic background and ongoing research focus on solid waste valorization and circular economy strategies. I work on sustainable solutions for the agro-industrial sector, particularly the transformation of waste streams into value-added products.

I confirm that I have read this submission and believe that I have an appropriate level of expertise to confirm that it is of an acceptable scientific standard, however I have significant reservations, as outlined above.

# Reviewer report: Reviewer response for version 2

**DOI:** 10.21956/openreseurope.21750.r52578

**Published:** 2025-04-04

**Authors:** Alev Kir

**Affiliations:** 1 Olive Research Institute (ORI), Izmir, Turkey

I confirm that I have no other comment for the article.

Is the rationale for developing the new method (or application) clearly explained?

Partly

Is the description of the method technically sound?

Yes

Are the conclusions about the method and its performance adequately supported by the findings presented in the article?

Yes

If any results are presented, are all the source data underlying the results available to ensure full reproducibility?

Yes

Are sufficient details provided to allow replication of the method development and its use by others?

Yes

Reviewer Expertise:

Conducts research in Agronomy on Organic annual and Olive in certified experimental open field of Institute’s and farmers' conditions. Involved a project (ORGANIC-PLUS-774340) funded by EU H2020 in 2018 and 'Coordinating Organic Plant Breeding Activities for Diversity' (COBRA-Core Organic II-Eranet) between 2013 and 2016. In 1990 has graduated from Ege Uni., Faculty of Agriculture, Soil Science and Plant Nutrition Dept. (Izmir, Turkey) and received MSc, PhD on Plant Nutrition and soil fertility. Researcher at MoAF (1994-continuing) and was head of the Organic Farming (OF) Department of Aegean Agricultural Research Institute (AARI, Izmir-Turkey) (2006-2017). Led three vegetable and legume research projects in the national organic farming program of Turkey. Currently works at the Department of Soil Science and Plant Nutrition at Olive Research Institute (ORI)(Izmir Turkey) and leaded 21 research projects and tasks.

I confirm that I have read this submission and believe that I have an appropriate level of expertise to confirm that it is of an acceptable scientific standard.

# Reviewer report: Reviewer response for version 1

**DOI:** 10.21956/openreseurope.21103.r51478

**Published:** 2025-03-19

**Authors:** Ramisah Mohd Shah

**Affiliations:** 1 Universiti Malaysia Terengganu, Terengganu, Malaysia

Review: 

 Abstract:

 1.    The methodology is briefly described, but the selection criteria (16) are not explained. A brief mention of what aspects they cover (e.g., environmental impact, cost, availability) would help.

 2.    "Promising circular fertilizers"—It would be helpful to clarify whether the selected options have already been tested in agriculture or are just theoretically promising.

 Introduction:

 1.    Introduction is well-structured, informative, and logically flows from the importance of fertilizers to the need for circular alternatives.

 2.    The description on why conventional fertilizers is problematic was included, but it would help to contrast their environmental impact with the benefits of circular fertilizers in a separate sentence for better emphasis.

 3.    Standardized the use of the word ‘fertilizers’ or ‘fertilisers’ throughout the article because they are mixed up.

 Methods:

 Methodology is well-structured and provides a clear approach for mapping and selecting circular fertilizer value chains.

 Results and Discussion:

 Minor Typos of citation: Bøen & Haraldsen,, 2013” → Extra comma and (Karimi et al., 2022) appeared 2 times (at the beginning and end of sentence).

 Conclusion

 The conclusion is comprehensive and well-structured, effectively summarizing the FER-PLAY methodology and its key findings.

Is the rationale for developing the new method (or application) clearly explained?

Yes

Is the description of the method technically sound?

Yes

Are the conclusions about the method and its performance adequately supported by the findings presented in the article?

Yes

If any results are presented, are all the source data underlying the results available to ensure full reproducibility?

Yes

Are sufficient details provided to allow replication of the method development and its use by others?

Yes

Reviewer Expertise:

Agronomy, Plant genetic resources

I confirm that I have read this submission and believe that I have an appropriate level of expertise to confirm that it is of an acceptable scientific standard.

# Reviewer report: Reviewer response for version 1

**DOI:** 10.21956/openreseurope.21103.r51474

**Published:** 2025-03-11

**Authors:** Alev Kir

**Affiliations:** 1 Olive Research Institute (ORI), Izmir, Turkey

The important points I would like to draw attention to regarding the article are listed below. Accordingly;

1. In the Abstract and Introduction sections, the article particularly emphasizes the overwhelming dominance of conventional fertilizers in use. Can we say that this information really carries an importance and necessity that serves the purpose of the article? Since my opinion is to the contrary, I suggest that this issue be considered.
2. I have criticisms of the terminology generally used in the article. For example, the term fertilizer used does not match the materials examined. There is a tendency to use the term "fertilizer" mostly in chemical products containing a high percentage of plant nutrients. The targeted and studied materials are not evaluated in this sense. I suggest that this issue be considered.
3. The evaluation of risky and toxic materials in the article in the same order as materials that do not pose a risk in terms of environmental health has resulted in the evaluation of completely different issues by subjecting them to a ranking.
4. I present to your attention the evaluation of materials such as bio-waste compost and manure under independent subheadings.

Is the rationale for developing the new method (or application) clearly explained?

Partly

Is the description of the method technically sound?

Yes

Are the conclusions about the method and its performance adequately supported by the findings presented in the article?

Yes

If any results are presented, are all the source data underlying the results available to ensure full reproducibility?

Yes

Are sufficient details provided to allow replication of the method development and its use by others?

Yes

Reviewer Expertise:

Conducts research in Agronomy on Organic annual and Olive in certified experimental open field of Institute’s and farmers' conditions. Involved a project (ORGANIC-PLUS-774340) funded by EU H2020 in 2018 and 'Coordinating Organic Plant Breeding Activities for Diversity' (COBRA-Core Organic II-Eranet) between 2013 and 2016. In 1990 has graduated from Ege Uni., Faculty of Agriculture, Soil Science and Plant Nutrition Dept. (Izmir, Turkey) and received MSc, PhD on Plant Nutrition and soil fertility. Researcher at MoAF (1994-continuing) and was head of the Organic Farming (OF) Department of Aegean Agricultural Research Institute (AARI, Izmir-Turkey) (2006-2017). Led three vegetable and legume research projects in the national organic farming program of Turkey. Currently works at the Department of Soil Science and Plant Nutrition at Olive Research Institute (ORI)(Izmir Turkey) and leaded 21 research projects and tasks.

I confirm that I have read this submission and believe that I have an appropriate level of expertise to confirm that it is of an acceptable scientific standard, however I have significant reservations, as outlined above.

# Response

**Published:** 2025-03-28

**Authors:** Lucía González

**Affiliations:** Galician Water Research Center Foundation, Spain

Thank you for reviewing our article and giving valuable feedback. We will address each comment by the number you presented.

1. We have changed the introduction section to shift the focus away from the use of conventional fertiliser and more into why circular alternatives are important.
2. Since this article is part of the outputs of the public funded project FER-PLAY the terminology used is the same that was agreed by the consortium and advisory board of this project. We added the  word “circular” to try and clarify that the focus is on the recovery of resources.
3. Both the exclusionary and evaluative criteria presented in this article were agreed upon based on a series of interactions with stakeholders of the projects such as fertilisers producers and farmers associations, academic and policy experts. As the most relevant aspect to consider when evaluating value chains to produce circular fertilisers.
4. The focus of this project was on resource recovery. Using existing secondary materials and giving them further use. Because of this, the classifications used are based on the origin and type of secondary raw material and not on the characteristics of the final circular fertiliser produced (such as being organic or mineral).
